# The cost-effectiveness of bevacizumab, ranibizumab and aflibercept for the treatment of age-related macular degeneration—A cost-effectiveness analysis from a societal perspective

**DOI:** 10.1371/journal.pone.0197670

**Published:** 2018-05-17

**Authors:** Freekje van Asten, Charlotte T. J. Michels, Carel B. Hoyng, Gert Jan van der Wilt, B. Jeroen Klevering, Maroeska M. Rovers, Janneke P. C. Grutters

**Affiliations:** 1 Department of Ophthalmology, Radboud University Medical Center, Nijmegen, The Netherlands; 2 Department for Health Evidence, Radboud University Medical Center, Nijmegen, The Netherlands; Centre For Eye Research Australia, AUSTRALIA

## Abstract

**Background:**

The discussion on the use of bevacizumab is still ongoing and often doctors are deterred from using bevacizumab due to legal or political issues. Bevacizumab is an effective, safe and inexpensive treatment option for neovascular age-related macular degeneration (AMD), albeit unregistered for the disease. Therefore, in some countries ophthalmologists use the equally effective but expensive drugs ranibizumab and aflibercept. We describe the economic consequences of this dilemma surrounding AMD treatment from a societal perspective.

**Methods:**

We modelled cost-effectiveness of treatment with ranibizumab (as-needed), aflibercept (bimonthly) and bevacizumab (as-needed). Effectiveness was estimated by systematic review and meta-analysis. The drug with the most favourable cost-effectiveness profile compared to bevacizumab was used for threshold analyses. First, we determined how much we overspend per injection. Second, we calculated the required effectiveness to justify the current price and the reasonable price for a drug leading to optimal vision. Finally, we estimated how much Europe overspends if bevacizumab is not first choice.

**Results:**

Bevacizumab treatment costs €27,087 per year, about €4,000 less than aflibercept and €6,000 less than ranibizumab. With similar effectiveness for all drugs as shown by meta-analysis, bevacizumab was the most cost-effective. Aflibercept was chosen for threshold analyses. Aflibercept costs €943 per injection, but we determined that the maximum price to be cost-effective is €533. Alternatively, at its current price, aflibercept should yield about twice the visual gain. Even when optimal vision can be achieved, the maximum price for any treatment is €37,453 per year. Most importantly, Europe overspends €335 million yearly on AMD treatment when choosing aflibercept over bevacizumab.

**Conclusion:**

Bevacizumab is the most cost-effective treatment for AMD, yet is not the standard of care across Europe. The registered drugs ranibizumab and aflibercept lead to large overspending without additional health benefits. Health authorities should consider taking steps to implement bevacizumab into clinical practice as first choice.

## Introduction

Bevacizumab is an inexpensive and effective anti-vascular endothelial growth factor (VEGF) drug for neovascular age-related macular degeneration (AMD). Yet, recently it was described how ophthalmologists in the UK are deterred from prescribing this drug.[1] Instead doctors are required to prescribe the much more expensive ranibizumab or aflibercept by the General Medical Council, which opposes the use of off-label drugs such as bevacizumab when registered drugs are available. Even the National Institute for Health and Care Excellence (NICE), which holds cost-effectiveness in high regard, recommends the registered drugs for AMD, and is unable to make recommendations regarding bevacizumab.[2, 3] The article by Cohen describes the many political and legal issues involved in the development of such guidelines that seem to disregard cost-effectiveness. This, however, does not occur solely in the UK. In fact, in many other European countries bevacizumab is not allowed for ophthalmological indications. Europe is overspending by not addressing this problem adequately as anti-VEGF treatment accounts for a large proportion of total health care expenditures.[4]

AMD is a major health concern, affecting almost 200 million people globally within the next five years. Over 11 million will have developed end-stage AMD and about 2.8 million will reside in Europe.[5] Two-thirds will be neovascular AMD cases, requiring multiple anti-VEGF treatments. In the neovascular end-stage of AMD, increased VEGF levels cause newly formed blood vessels to grow into the retina and leak fluid, lipids and proteins, leading to rapid and severe vision loss. Anti-VEGF agents that can be injected in the eye have been developed to inhibit these neovascularisations and with great success. Pivotal randomized controlled trials (RCT) have shown the superiority of anti-VEGF treatment over placebo improving both vision and quality of life in general.[6–8] Now, anti-VEGF therapy has become the mainstay of neovascular AMD treatment. Treatment in clinical practices worldwide is dominated by three anti-VEGF agents: ranibizumab, aflibercept and bevacizumab.

Ranibizumab (Lucentis, Genentech Inc./Novartis) and aflibercept (Eylea, Bayer) are both registered for neovascular AMD. A large non-inferiority RCT comparing aflibercept and ranibizumab showed no discernible difference in effectiveness.[9] Bevacizumab (Avastin, Genentech Inc./Roche) is not registered for use in AMD. The property rights of bevacizumab and ranibizumab are both owned by Roche, the parent company of Genentech, and although bevacizumab was originally developed as a cancer treatment, it has similar properties as ranibizumab, both being VEGF-A antibodies. RCTs have shown that there is no meaningful difference between the two agents in effect on visual acuity.[10–13] However, although the three agents are equally effective in conserving vision, there are large differences in costs. Bevacizumab is a factor 20 less expensive than ranibizumab and aflibercept, i.e. about €50 and €1,000 per injection. As the EU member states are aiming towards containment of expenditures to health care and efficient allocation of resources one would expect that bevacizumab would be first choice in AMD treatment. However, wide differences exist across Europe in rules and legislations surrounding the use of this off-label drug and so its use in clinical practice is not self-evident.

In this article, we describe how much we are overspending on registered anti-VEGF drugs and we provide the economic arguments for AMD treatment decisions, pricing and innovation. We provide insight into the costs involved in ranibizumab, aflibercept and bevacizumab treatment and show the economic consequences of not allowing bevacizumab in ophthalmology. Supported by a cost-effectiveness analysis we will discuss why bevacizumab is a reasonable first line treatment in AMD from a public health perspective.

## Methods

### Model development

To estimate the economic consequences for society of prescribing ranibizumab or aflibercept instead of bevacizumab we developed a patient-level decision analytic model evaluating effectiveness, quality of life and costs. Three treatment strategies were compared: ranibizumab administered as-needed, aflibercept administered bimonthly and bevacizumab administered as-needed. The as-needed regimen includes a loading phase of three consecutive monthly injections, followed by injections on an as-needed basis, depending on clinical evaluation. The bimonthly regimen also includes a loading phase, followed by injections every two months. These regimens reflect daily care in clinical practice most closely, but are also supported by evidence from RCTs. We omitted a “no treatment” option as past studies showed anti-VEGFs were cost-effective in comparison to past treatments.[14] We therefore deemed withholding anti-VEGF from patients to be undesirable and did not consider this option.

The model estimated the mean costs and benefits for a hypothetical group of patients. AMD patients with a mean (SD) baseline visual acuity of 52.1 (14.1) entered the model individually, were cloned into 3 identical patients and each of these three patients progressed through one of the three treatment strategies in the model over one year. Each patient was assigned a baseline visual acuity and change in visual acuity for each of the treatment strategies using a normal distribution based on the mean and SD values listed in Table 1. The outcomes were averaged across a large sample of patients (n = 100). We employed a 1-year time horizon since this time span is most extensively studied and allows for the most accurate effect estimates. Because of this relatively short time frame we did not apply discounting. The model assumed treatment of the best seeing eye, which is known to be most strongly predictive of quality of life.[8, 15]

**Table 1:** Results of meta-analysis.

Variable		
**Baseline visual acuity (ETDRS letters) mean (SD)**	52.1	(14.1)
**Change in visual acuity (ETDRS letters) mean (SD)**		
Ranibizumab	4.7	(15.9)
Aflibercept	8.4	(14.7)
Bevacizumab	5.4	(8.8)
**Number of injections**		
Ranibizumab	6.2	
Aflibercept	7.0	
Bevacizumab	7.0	

ETDRS = early treatment for diabetic retinopathy study; SD = standard deviation.

### Effectiveness and quality of life

To determine the effectiveness of each drug, a systematic review and meta-analysis was conducted. We searched Pubmed, Embase, and the Cochrane Library from inception to 12 May 2014 with the terms: age-related macular degeneration, aflibercept, bevacizumab, ranibizumab, visual acuity, and optical coherence tomography (see S1 Table for full search strategy). This search yielded 438 publications. Studies were included if they met the following inclusion criteria: (I) RCT phase III / IV, (II) follow-up period of at least 1 year, (III) patients diagnosed with neovascular age-related macular degeneration, (IV) compared treatments with aflibercept, bevacizumab, ranibizumab or placebo (V) reported on change in visual acuity effectiveness outcome. Study populations of predominately Asian patients (>25%), non-treatment naïve patients, and studies that only investigated switching patients to other dosages and treatments were excluded. For ranibizumab and bevacizumab only RCTs reporting results for as-needed regimens for the drugs separately were included, while for aflibercept only bimonthly regimens were considered. Finally, seven RCTs were included (S2 Table).[9–13, 16, 17] The results are presented in Table 1. Data were pooled with a random effects meta-analysis. Statistical analyses were performed using RevMan5.2 (The Nordic Cochrane Centre). Because the comparisons were incomplete (no direct comparisons for aflibercept bimonthly versus ranibizumab or bevacizumab as-needed), we calculated weighted means and standard deviations for each treatment arm separately. Effectiveness was defined as change in visual acuity from baseline in ‘early treatment for diabetic retinopathy study’ (ETDRS) letters after 1 year of treatment. ETDRS letters are scored on a letter chart with letters of decreasing size from top to bottom. The number of letters a patient can read on the letter chart determines the visual acuity. Baseline visual acuity was derived from a meta-analysis of the baseline ETDRS letter scores of all included RCTs.

Based on their visual acuity after 1 year of treatment, patients were assigned a utility score which was derived from literature (Table 2).[18] This four-level classification is one of the most widely used classifications and more importantly, distinguishes clinically meaningful subgroups. We assumed that this utility score increased immediately after the start of treatment and remained constant throughout the 1-year time horizon of the model. Utility scores represent the quality of life of a patient with a certain health status, or in this case with a certain visual acuity score. Utility scores vary from 1 to 0, with 1 indicating a perfect health state and 0 indicating death. The use of utility scores allows the calculation of quality adjusted life years (QALYs). One QALY equals one year lived in perfect health.

**Table 2:** Utility value per visual acuity category.

Visual acuity category	Visual acuity range	Utility value	Standard error	Distribution
1.	20/20-20/25	0.84	0.027	Beta
2.	20/30-20/40	0.80	0.024	Beta
3.	20/50-20/100	0.71	0.029	Beta
4.	≤ 20/200	0.59	0.027	Beta

Derived from Brown et al. 2002[18]

### Costs

Costs were calculated from a societal perspective, including both direct and indirect costs. Direct medical costs included the costs of the drugs, medical visits and ophthalmic examinations. Drug costs were obtained from the Dutch National Health Care Institute[19] and costs for ophthalmic examinations were obtained from the department of Ophthalmology of the Radboudumc. Notably, the costs for bevacizumab are specifically per injection and not per vial. The mean number of injections for ranibizumab and bevacizumab in a year was estimated from published RCTs by calculating the weighted average. The number of injections for aflibercept was set at 7, corresponding with a bimonthly regimen. Number of injections was considered fixed. Indirect costs included the costs of low vision aids and nonmedical costs. The costs of low vision aids were based on a database from the Low Vision Totaal vision clinic containing 550 patients with various forms of maculopathy and their use of vision aids. Per visual acuity category the mean costs for low vision aids per person were calculated. Nonmedical costs, including caregiver costs, adapted housing and transportation, were derived from literature.[20] S3 Table provides an overview of all estimated costs. Costs are estimated in 2014 euro (€) and adjusted for inflation, using index prices according to the Dutch pharmaco-economic guidelines[21] and presented in euro or equivalent pound (£) when appropriate (currency rate €/£ January 1^st^ 2015: 0.77661).

### Analysis

We developed the decision analytic model using ARENA software (version 14.00.00 Rockwell Automation, Inc). The three treatments were compared in terms of mean costs, mean effects (in QALYs) and incremental cost-effectiveness ratios (ICERs). The ICER represents the extra costs that are incurred in order to gain one additional QALY, comparing one treatment over the other. The ICER is calculated by dividing the estimated difference in costs between two treatments by the difference in QALYs.[22]

Whether a treatment may be considered cost-effective depends on how much a society would be willing to pay for a QALY. We used a willingness-to-pay (WTP) threshold of €80,000 per QALY, the highest cost-effectiveness threshold recommended by The National Health Care Institute.[23] This means that a strategy is deemed cost-effective compared to another strategy when it costs €80,000 or less to gain an extra QALY, i.e. the ICER is lower than €80,000 per QALY. Bevacizumab was used as the comparator in all cases. Probabilistic sensitivity analyses were performed to assess the impact of uncertainty on the outcomes.[24] This means that we assigned distributions to model parameters, to reflect the uncertainty in the estimation of that parameter when possible. All distributions are listed in Tables 1 and 2 and S3 Table. Parameter values were sampled at random from the assigned distributions, using Monte Carlo simulation. After each sample, 100 patients were simulated using these parameter values, which was repeated 5000 times. This was sufficient to reach convergence. Cost-effectiveness acceptability curves were plotted, which show the probability that an anti-VEGF drug is the most cost-effective over a range of WTP thresholds.

To answer our question of what an acceptable price is for innovative treatments in AMD, we performed threshold analyses for the drug with the most favourable cost-effectiveness profile compared to bevacizumab. Threshold analyses determine under what conditions the drug will become the most cost-effective treatment compared to bevacizumab, i.e. under which circumstances the ICER will drop below the WTP threshold of €80,000 per QALY. First, the acceptable price for one injection of the currently available drug was calculated. Given the effectiveness of the drug, we calculated up to which drug costs the ICER was below €80,000. These total maximum drug costs were then divided by the total number of injections to determine the acceptable price per injection. Second, we determined how effective the drug should be to justify the difference in costs compared to bevacizumab. For this analysis, we kept the costs stable, but we varied the change in visual acuity until the resulting quality of life gain was sufficient to reach the WTP threshold of €80,000. Third, we calculated the acceptable price for a theoretical innovative treatment that leads to optimal visual acuity. For the calculation with optimal visual acuity, we assumed that all patients would reach the highest utility value as reported in Table 2 and determined what price of this theoretical drug resulted in an ICER of €80,000. When varying quality of life gains, indirect costs were reduced as quality of life gains increased. The reduction in indirect costs per 0.01 QALY was estimated through linear regression in the modelled patient population using the model output. Similarly, we used linear regression to determine the number of QALYs gained per ETDRS letter to translate quality of life changes to visual acuity changes.

Overspending was defined as the amount of extra money paid for health care that cannot be justified by the WTP threshold of €80,000, i.e. additional treatment costs without an equivalent health benefit. As opposed to the absolute price difference, the amount of overspending is based on the difference between the current price and the cost-effective price and therefore represents the health care budget that is wasted. Overspending in Europe was calculated based on the overpricing of the registered drug compared to bevacizumab and the number of expected injections. The number of injections in the UK was derived from incidence data of new patients requiring treatment for neovascular AMD (102 per 100,000)[25] and the assumption that most patients in real-world clinical practice will receive on average 5 injections in the first year.[26–29] We assumed that treating all patients with bevacizumab would be unfeasible considering some patients may be non-responders requiring secondary treatment. From the department of ophthalmology of the Radboudumc, where bevacizumab is first choice, we could estimate that 80% of injections for AMD are with bevacizumab, while the other 20% are mostly with aflibercept reserved for non-responders. All estimates were then extrapolated to the entire European Union, which has a population of approximately 200 million aged over 50 years.[30]

Reporting in this economic evaluation was based on the Consolidated Health Economic Evaluation Reporting Standards (CHEERS) statement.[31]

## Results

### Cost-effectiveness

**Fig 1:**
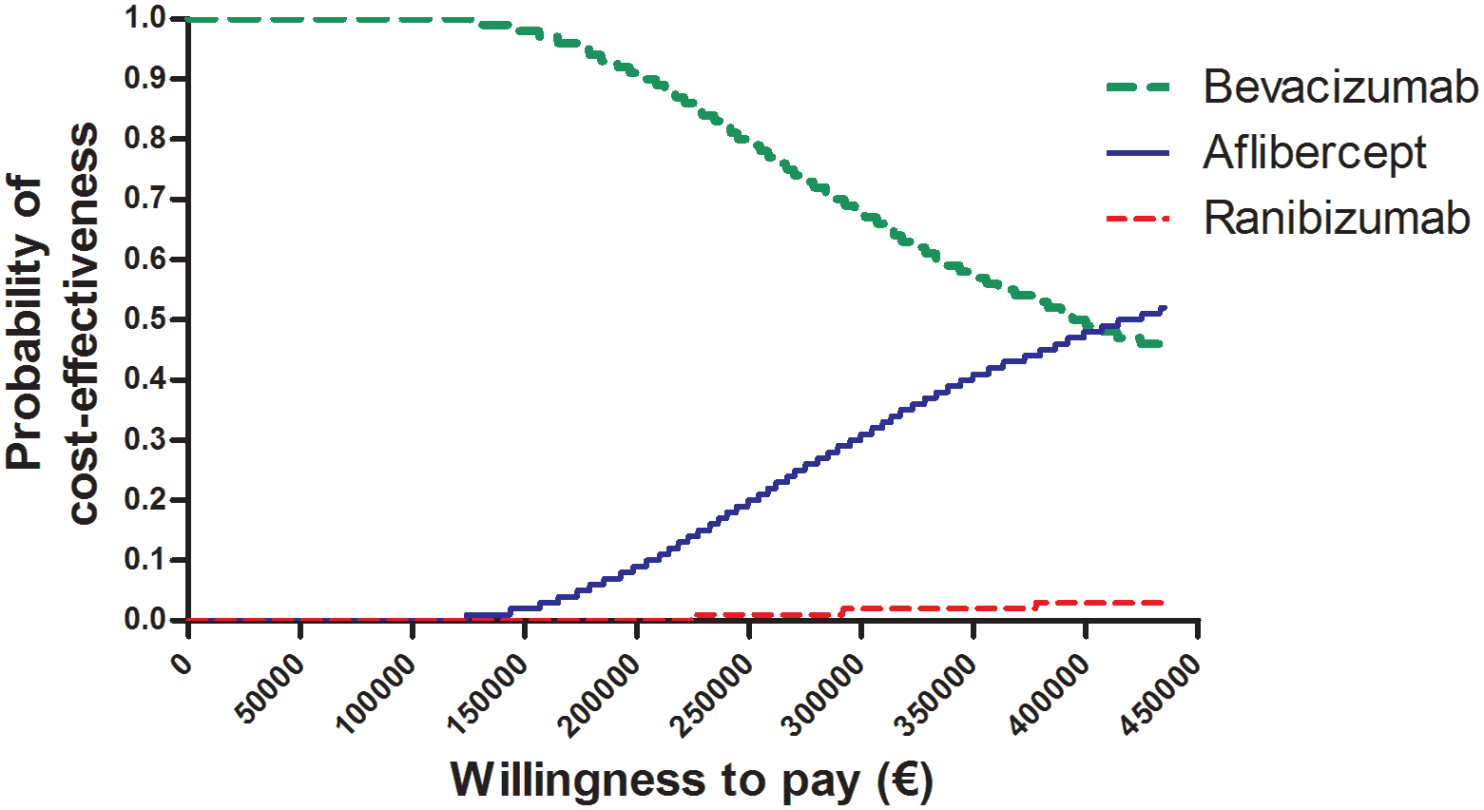
Acceptability curve of the three anti-VEGF treatments. The acceptability curve shows the probability of a treatment being cost-effective over a range of willingness-to-pay thresholds. The curve shows that bevacizumab is the most likely to be cost-effective until a willingness-to-pay threshold of €407,250 is reached, after which aflibercept is most likely to be cost-effective.

Ranibizumab was dominated by bevacizumab in the cost-effectiveness model, meaning that bevacizumab costs less than ranibizumab and was more or equally effective. Aflibercept yielded 0.015 extra QALYs compared to bevacizumab. Assuming a threshold of €80,000 for one QALY gained, bevacizumab was the most cost-effective treatment in 100% of simulations (Fig 1). Using aflibercept instead of bevacizumab means paying €278,099 per QALY gained (Table 3). Using ranibizumab would mean spending money without any health benefits over bevacizumab.

**Table 3:** Differences in effectiveness and costs between treatments, per patient in the first year of treatment. The ICERs show that we pay €278,099 per QALY if we use aflibercept instead of bevacizumab and that ranibizumab is dominated by bevacizumab, meaning it is costlier, but does not yield health benefit.

	Mean costs € (95-%CI)	Difference in costs Δ€	Mean effectiveness QALY (95-%CI)	Difference in effectiveness ΔQALY	ICER Δ€/ΔQALY
**Ranibizumab**	€ 33,137 (28,883–37,926)	€ 6,050	0.69 (0.66–0.73)	0.000	Dominated
**Aflibercept**	€ 31,119 (26,979–35,766)	€ 4,032	0.71 (0.67–0.74)	0.015	€ 278,099
Bevacizumab*	€ 27,087 (22,818–31,789)	-	0.69 (0.66–0.73)	-	-

QALY = quality-adjusted life year; ICER = incremental costs-effectiveness ratio; 95%-CI = 95%-confidence interval;

*Bevacizumab is comparator.

### Threshold analyses

**Fig 2:**
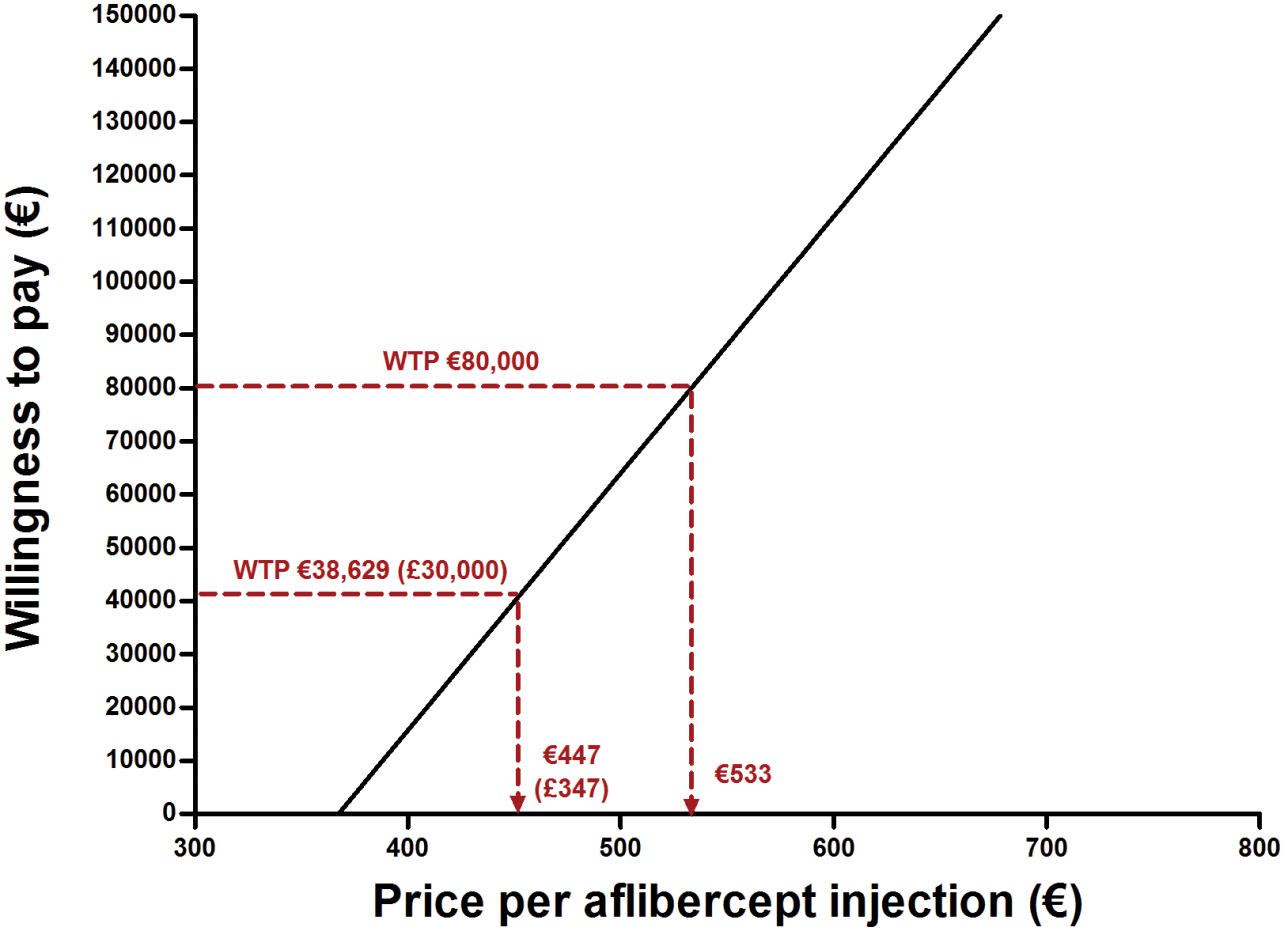
Acceptable price per aflibercept injection. Which price is acceptable for one aflibercept injection depends on what society is willing to pay. The graph shows that at a willingness-to-pay of €80,000 per QALY aflibercept should cost no more than €533. To reach the NICE threshold for cost-effectiveness, the costs should be reduced to £347.

Because aflibercept was more promising in terms of cost-effectiveness, we chose this drug to show what would be necessary in order for it to be a justifiable alternative to bevacizumab. The current acquisition costs of aflibercept are €943. For aflibercept to meet the standard criteria of cost-effectiveness, this should be reduced to €533 per injection. This means aflibercept is currently €410 overpriced. Because WTP thresholds may vary across countries, the relation between the theoretically acceptable price for one aflibercept injection and WTP is shown in Fig 2. At a willingness to pay of £30,000 (€38,629), the threshold practised by NICE, aflibercept should cost no more than £347 (€447) per injection.

We also reversed the question by estimating how well aflibercept should work to justify the current difference in cost with bevacizumab. The difference in total costs between aflibercept and bevacizumab was €4,032 per patient in the first year. Taking into account that indirect costs will decrease as effectiveness increases, we calculated that aflibercept should at least render an additional 0.041 QALYs per patient compared to bevacizumab. Linear regression showed that 0.004 QALYs corresponded to approximately 1 letter on ETDRS chart. This implies that aflibercept should at least render an additional visual gain of 10.3 letters compared to bevacizumab, which is a change from baseline of 15.7 letters instead of the current 8.4 letters.

New drugs are constantly being developed and one day these may be highly effective in restoring vision. The maximum utility to be reached in neovascular AMD was established to be 0.84 in the highest visual acuity group.[18] Bevacizumab treatment leads to a mean utility score of 0.69, implying that at maximum 0.15 QALYs can be gained each year. To remain within the €80,000 WTP threshold, total costs for the new treatment should not exceed €38,813 during the first year. Since indirect costs for this new treatment will be lower when effectiveness increases, we re-calculated the indirect costs to be €1,360 (S3 Table) when vision is optimally restored. After subtracting indirect costs, the direct medical costs of a treatment that accomplishes perfect vision in AMD should not exceed €37,453 per year.

### Financial consequences

In the UK alone the number of new cases with neovascular AMD requiring treatment will rise to 32,000 within the next 5 years.[25] In our calculations we assumed 7 bevacizumab and aflibercept injections, however, usually in clinical practice we tend to administer fewer.[26–29] More likely, these patients will receive on average 5 injections in their first year, summing up to 160,000 injections in total. The UK does not have bevacizumab as their first-choice treatment since the NICE guidelines recommend the registered drugs instead. Assuming that 80% of patients could be treated with bevacizumab instead, we have calculated that the UK is overspending over €52.5 million (£38.7 million) each year on new neovascular AMD patients. For Europe, we estimate that approximately 204,000 new AMD patients will require roughly 1 million injections. Treating 80% of these people with bevacizumab instead of aflibercept, would save Europe approximately €335 million yearly. Repeating the same calculations assuming 7 injections of aflibercept, these numbers become €73.5 million (£57.1 million) for the UK and €468 million for Europe.

## Discussion

Bevacizumab is the most cost-effective treatment for neovascular AMD. That aflibercept and ranibizumab are not cost-effective compared to bevacizumab does not come as a surprise. Several studies based on RCT data have been performed showing that bevacizumab is the most cost-effective in comparison to ranibizumab[32–34] and aflibercept.[35, 36] The majority of studies are based on at most two RCTs[32–34, 36] and many lack an estimated societal impact and economic consequences, especially with regard to aflibercept. With limited health care budgets, it is important to present realistic estimates of spending. We estimated that each injection of aflibercept is €410 overpriced and that Europe overspends €335 million on health care if aflibercept is first-choice treatment instead of bevacizumab. The overspending of implementing ranibizumab as first-choice will be even bigger. An important remark here is that these costs are already adjusted for any added health benefit from the registered drugs, which was unsubstantial. Visual acuity gains of aflibercept were estimated to be approximately 3 letters compared to bevacizumab, corresponding to 0.01 QALYs. A difference this small will not be perceived by the individual patient.[37]

The major strength of our model is that it provides a clear view of the total costs involved in choosing one drug over the other. This study presents the absolute overspending associated with aflibercept treatment and can thus contribute to budgetary discussions as well as negotiations between government and pharmaceutical companies. Some possible limitations should also be discussed. First, we assumed treatment of the best-seeing eye in our model since we know that the vision related quality of life in an individual is mostly determined by the vision in the eye that sees best and treating the worst-seeing eye will have little impact on quality of life.[8] Modelling the best-seeing eye will therefore lead to conservative estimates, and thus cost-effectiveness is probably even poorer in real-life. Second, we did not consider adverse events of any kind. A recent Cochrane review evaluated the evidence for systemic safety of ranibizumab and bevacizumab (n = 3,665) and they found no relevant differences except for gastrointestinal events.[38] Interestingly, the authors recommend that health care authorities refrain from prohibiting bevacizumab on account of theoretical safety issues. Evidence for safety of aflibercept is still lagging behind, but in the large RCT of aflibercept versus ranibizumab no differences in safety were noted.[9] Third, medical costs were from a Dutch source and nonmedical costs were derived from an American source. It is unsure whether these are fully representative of the European setting. We do note, however, that because differences in effectiveness are extremely small and the difference in drug costs is extremely large, the largest effect on cost-effectiveness is determined by drug costs and not by other costs. Nonmedical costs may differ from country to country, but the drug costs are more similar. Nevertheless, the larger the cost differences between vision categories, the more favourable the cost-effectiveness of aflibercept becomes. When the cost differences between the vision categories become bigger, small differences in effectiveness become more valuable, so there will be some variation in results depending on how you estimate nonmedical costs. In our model, the cost differences were substantial. Nonetheless, aflibercept did not come close to being cost-effective. Fourth, we only estimated costs and effects for the first year of treatment. After this year, patients are still treated, but there is insufficient evidence for the number of injections and the effectiveness hereafter. Arguably, in the following years costs are still made, but vision is expected to deteriorate. Hence, the actual overspending is probably underestimated. It is recommendable to repeat this cost-effectiveness analysis in the future when more effectiveness data becomes available and with long-term follow-up. Possibly, a network meta-analysis could improve some of our estimates, although previous attempts have not shown results very different from ours, with aflibercept resulting in 1.3% more letters gained in comparison to bevacizumab.[39] Several underlying assumptions of our model have been discussed and it should be realized that changes in assumptions and in model input may affect end-results. For example, health care costs may vary across countries and may change over time. A simulation model has limitations that are inherent to the model itself. It is based on best-available evidence and is therefore dependent on the quality of this data and the certainty around it. Small changes may have large effects as discussed above. This cost-effectiveness model should be updated as more robust evidence is published. Despite all limitations mentioned, it is clear that ranibizumab and aflibercept are grossly overpriced.

Europe is divided when it comes to the use of off-label drugs. As we know now, the General Medical Council and the NICE guidelines in the UK dissuade ophthalmologists from using bevacizumab, as it lacks proper appraisal. Unfortunately, the UK is not the only country where the use of this cost-effective alternative is held back by legislation. In countries such as Germany, Switzerland, Belgium, and until recently France, the use of bevacizumab in ophthalmology is generally not prescribed because the health authorities declare that off-label use of drugs should be avoided when a registered alternative is available. However, in the case of bevacizumab, should there not be an exception seeing the immense benefits?

The discussion here is not only about overpaying for a drug when a less costly alternative is available. Some may argue that even a little gain in quality of life is reason enough to use one drug over the other. This seems ethical, but is actually the opposite. Health care funds are not an infinite resource and investing in one part of health care, means having less resources left for other types of health care. For the National Health Services (NHS) in the UK this has been meticulously researched by Claxton et al.[40, 41] They published new methods for estimating NICE cost-effectiveness thresholds which was accompanied by a calculator to estimate the overall loss of QALY based on the additional costs of approving a new technology. A year’s worth of aflibercept treatment costs €4,032 more than bevacizumab, which means an additional investment of €129 million to treat the AMD-population in the UK. The calculator shows us that this additional investment requires a redistribution of health care, resulting in a total of 7,346 QALYs lost because of a reduction of resources in other types of health such as respiratory diseases and mental health. Therefore, not only does the use of aflibercept over bevacizumab lack a substantial health benefit, it in fact may result in loss of overall health within a society.

A simple solution would seem to register bevacizumab for use in AMD. However, only Roche, who has the ownership rights to bevacizumab, can request such a registration and Roche also owns its costlier alternative ranibizumab. Roche states that bevacizumab is not manufactured or approved for intraocular use and they wish to focus on developing bevacizumab further for oncological indications and not for ophthalmology. However, we cannot rely on commercial companies to actively contribute to financial sustainability of health care and this should be the task of the health care authorities. The registration and patent policies will probably not change anywhere in the near future, but ranibizumab’s patent will be ending in 2018[42] and if not extended, this could hopefully lead to an influx of less costly generic alternatives.

In conclusion, AMD is a prevalent and debilitating disease with a large demand for treatments. Value based pricing in health care means that drugs should be priced in accordance with their health benefits.[43] It is clear that aflibercept and ranibizumab do not reach these criteria. New drugs are continuously being designed and as long as policy makers are negligent of cost-effectiveness and sustainability of health care, drugs can be marketed at unaffordable prices. The question remains whether the development of these new agents is truly justified, considering the availability of the inexpensive and effective bevacizumab. Developing a new drug at the cost of below €40,000 per year will be a challenging task. The awareness of the importance of cost-effectiveness to keep health care sustainable is growing and new drugs should be critically appraised on their added value. In the case of neovascular AMD treatment there is no question; bevacizumab is the most cost-effective anti-VEGF treatment for AMD.

## Supporting information

S1 Table Search strategy for systematic review of randomized controlled trials on anti-VEGF therapy in neovascular age-related macular degeneration. (PDF)

S2 Table Randomized controlled trials used to estimate treatment effect. (PDF)

S3 Table Direct and indirect costs for neovascular AMD treatment. (PDF)
