# Supplementary material for: The cost-effectiveness of bevacizumab, ranibizumab and aflibercept for the treatment of age-related macular degeneration—A cost-effectiveness analysis from a societal perspective
Source: PLoS One. 2018 May 17;13(5):e0197670. doi: 10.1371/journal.pone.0197670 (PMC5957378; doi:10.1371/journal.pone.0197670)
Supplement: S1 Table — (PDF) [file pone.0197670.s001.pdf]

S1 Table. Search strategy for systematic review of randomized controlled trials on anti-VEGF therapy in neovascular age-related macular degeneration

**Pubmed database search on 12 May 2014**		
**Terms**	**Syntax**	**Number of records**
Effectiveness	Comparative effectiveness research[MeSH] OR evaluation studies as topic[MeSH] OR cost-benefit analysis[MeSH] OR visual acuity[MeSH Terms] OR tomography, optical coherence[Mesh] OR effect[tiab] OR effects[tiab] OR effectiveness [tiab] OR value[tiab] OR value-based[tiab] OR benefit[tiab] OR benefits[tiab] OR evidence[tiab] OR evidence-based[tiab] OR visual acuity[tiab] OR visus[tiab] OR BCVA[tiab] OR OCT[tiab] OR optical coherence tomography[tiab]	6.279.175
Macular degeneration	wet macular degeneration[Mesh] OR macular degeneration[Mesh:NoExp] OR retinal degeneration[Mesh:NoExp] OR ((age-related[tiab] OR age related[tiab]) AND (macul*[tiab] OR retina*[tiab]) AND (degener*[tiab] OR neovascul*[tiab])) OR (exudative AND (macul*[tiab] OR retina*[tiab]) AND (degener*[tiab] OR neovascul*[tiab])) OR AMD[tiab] OR AMDs[tiab] OR ARMD[tiab] OR ARMDs[tiab]	23.567
Anti-VEGF	ranibizumab[Supplementary Concept] OR ranibizumab[tiab] OR lucentis[tiab] OR bevacizumab[Supplementary Concept] OR bevacizumab[tiab] OR avastin[tiab] OR aflibercept[Supplementary Concept] OR aflibercept[tiab] OR EYLEA[tiab] OR VEGF-trap[tiab] OR VEGF trap[tiab] OR vascular endothelial growth factor trap[tiab] OR angiogenesis inhibitors[Mesh] OR anti-VEGF[tiab] OR anti VEGF[tiab] OR anti vascular endothelial growth factor*[tiab] OR anti-vascular endothelial growth factor*[tiab] OR antiangiogenic*[tiab] OR anti-angiogenic*[tiab] OR intravitreal[tiab] OR VEGF inhibitor*[tiab] OR VEGF-inhibitor*[tiab]	37.279
Effectiveness **AND** Macular degeneration **AND** Anti-VEGF **AND** (Therapy/broad[filter]) AND (randomized controlled trial)	(comparative effectiveness research[MeSH] OR evaluation studies as topic[MeSH] OR cost-benefit analysis[MeSH] OR visual acuity[MeSH Terms] OR tomography, optical coherence[Mesh] OR effect[tiab] OR effects[tiab] OR effectiveness [tiab] OR value[tiab] OR value-based[tiab] OR benefit[tiab] OR benefits[tiab] OR evidence[tiab] OR evidence-based[tiab] OR visual acuity[tiab] OR visus[tiab] OR BCVA[tiab] OR OCT[tiab] OR optical coherence tomography[tiab]) **AND** (wet macular degeneration[Mesh] OR macular degeneration[Mesh:NoExp] OR retinal degeneration[Mesh:NoExp] OR ((age-related[tiab] OR age related[tiab]) AND (macul*[tiab] OR retina*[tiab]) AND (degener*[tiab] OR neovascul*[tiab])) OR (exudative AND (macul*[tiab] OR retina*[tiab]) AND (degener*[tiab] OR neovascul*[tiab])) OR AMD[tiab] OR AMDs[tiab] OR ARMD[tiab] OR ARMDs[tiab]) **AND** (ranibizumab[Supplementary Concept] OR ranibizumab[tiab] OR lucentis[tiab] OR	**224**

	bevacizumab[Supplementary Concept] OR bevacizumab[tiab] OR avastin[tiab] OR aflibercept[Supplementary Concept] OR aflibercept[tiab] OR EYLEA[tiab] OR VEGF-trap[tiab] OR VEGF trap[tiab] OR vascular endothelial growth factor trap[tiab] OR angiogenesis inhibitors[Mesh] OR anti-VEGF[tiab] OR anti VEGF[tiab] OR anti vascular endothelial growth factor*[tiab] OR anti-vascular endothelial growth factor*[tiab] OR antiangiogenic*[tiab] OR anti-angiogenic*[tiab] OR intravitreal[tiab] OR VEGF inhibitor*[tiab] OR VEGF-inhibitor*[tiab]) **AND** ((Therapy/Broad[filter]) AND (randomized controlled trial)) Filters: English	

**EMBASE database search on 12 May 2014**		
**Terms**	**Syntax**	**Number of records**
Effectiveness	exp comparative effectiveness/ OR exp evaluation/ OR exp cost benefit analysis/ OR exp visual acuity/ OR exp optical coherence tomography/ OR effect.ti,ab. OR effects.ti,ab. OR effectiveness.ti,ab. OR value.ti,ab. OR value-based.ti,ab. OR benefit.ti,ab. OR benefits.ti,ab. OR evidence.ti,ab. OR evidence-based.ti,ab. OR visual acuity.ti,ab. OR visus.ti,ab. OR BCVA.ti,ab. OR OCT.ti,ab. OR optical coherence tomography.ti,ab.	6.628.825
Macular degeneration	.exp exudative macular degeneration/ or exp retina macula age related degeneration/ or ((age-related.ti,ab. or age related.ti,ab.) and (macul*.ti,ab. or retina*.ti,ab.) and (degener*.ti,ab. or neovascul*.ti,ab.)) or (exudative.ti,ab. and (macul*.ti,ab. or retina*.ti,ab.) and (degener*.ti,ab. or neovascul*.ti,ab.)) or AMD.ti,ab. or AMDs.ti,ab. or ARMD.ti,ab. OR ARMDsti,ab.	18.943
Anti-VEGF	exp ranibizumab/ or ranibizumab.ti,ab. or lucentis.ti,ab. or exp bevacizumab/ or bevacizumab.ti,ab. or avastin.ti,ab. or exp aflibercept/ or aflibercept.ti,ab. or EYLEA.ti,ab. or VEGF-trap.ti,ab. or VEGF trap.ti,ab. or vascular endothelial growth factor trap.ti,ab. or exp angiogenesis inhibitors/ or anti-VEGF.ti,ab. or anti VEGF.ti,ab. or anti vascular endothelial growth factor*.ti,ab. or anti-vascular endothelial growth factor*.ti,ab. or antiangiogenic*.ti,ab. or anti-angiogenic*.ti,ab. or intravitreal.ti,ab. or VEGF inhibitor*.ti,ab. or VEGF-inhibitor*.ti,ab.	97.579
Effectiveness **AND** Macular degeneration **AND** Anti-VEGF		2.741
Filters: English and RCT		**164**

**Cochrane Library database search on 12 May 2014**		
**Terms**	**Syntax**	**Number of records**

Effectiveness	comparative effectiveness research[MeSH] or evaluation studies as topic[MeSH] or cost-benefit analysis[MeSH] or visual acuity[MeSH Terms] or tomography, optical coherence[Mesh] or (effect OR effects or effectiveness or value or value-based or benefit or benefits or evidence or evidence-based or visual acuity or visus or BCVA or OCT or optical coherence tomography):ti,ab,kw	490.744
Macular degeneration	wet macular degeneration[Mesh] or macular degeneration[Mesh:NoExp] or retinal degeneration[Mesh:NoExp] or (((age-related or age related) and (macul* or retina*) and (degener* or neovascul*)) or (exudative and (macul* or retina*) and (degener* or neovascul*)) or AMD or AMDs or ARMD or ARMDs):ti,ab,kw	1.629
Anti-VEGF	angiogenesis inhibitors[Mesh] or (ranibizumab or lucentis or bevacizumab or avastin or aflibercept or EYLEA or VEGF-trap or VEGF trap or vascular endothelial growth factor trap or anti-VEGF or anti VEGF or anti vascular endothelial growth factor* or anti-vascular endothelial growth factor* or antiangiogenic* or anti-angiogenic* or intravitreal or VEGF inhibitor* or VEGF-inhibitor*):ti,ab,kw	2.373
Effectiveness **AND** Macular degeneration **AND** Anti-VEGF		**384**
