# Supplementary material for: The cost-effectiveness of bevacizumab, ranibizumab and aflibercept for the treatment of age-related macular degeneration—A cost-effectiveness analysis from a societal perspective
Source: PLoS One. 2018 May 17;13(5):e0197670. doi: 10.1371/journal.pone.0197670 (PMC5957378; doi:10.1371/journal.pone.0197670)
Supplement: S2 Table — (PDF) [file pone.0197670.s002.pdf]

S2 Table. Randomized controlled trials used to estimate treatment effect

Study	Drug	Regimen	Number of patients	Mean number of injections	VA at baseline (ETDRS letters) mean (± SD)	Change in VA after 1 year (ETDRS letters) mean (± SD)	Reference
**CATT 2012**	Ranibizumab 0.5 mg	PRN	285	6.9	61.5 (13.2)	6.8 (13.1)	[10]
**Subramanian 2010**	Ranibizumab 0.5 mg	PRN	7	4.0	32.7 (20.9)	6.3 (13.7)	[11]
**Krebs 2013**	Ranibizumab 0.5 mg	PRN	263	5.8	56.4 (13.5)	4.1 (7.0)	[12]
**Kodjikian 2013**	Ranibizumab 0.5 mg	PRN	183	6.5	55.78 (13.99)	2.9 (15.1)	[13]
**Busbee 2013**	Ranibizumab 0.5 mg	PRN	275	7.7	54.5 (11.7)	8.2 (13.3)	[16]
**SAILOR 1 2009**	Ranibizumab 0.5 mg	PRN	490	4.9	48.9 (13.8)	2.3 (22.1)	[17]
**CATT 2012**	Bevacizumab 1.25 mg	PRN	271	7.7	60.4 (13.4)	5.9 (15.7)	[10]
**Subramanian 2010**	Bevacizumab 1.25 mg	PRN	15	8.0	34.93 (14.58)	7.6 (15.3)	[11]
**Krebs 2013**	Bevacizumab 1.25 mg	PRN	154	6.1	57.0 (13.0)	4.9 (7.0)	[12]
**Kodjikian 2013**	Bevacizumab 1.25 mg	PRN	191	6.8	54.62 (14.07)	4.8 (14.9)	[13]
**VIEW 1 2012**	Aflibercept 2 mg	Bimonthly	301	7	55.7 (12.8)	7.9 (15.0)	[9]
**VIEW 2 2012**	Aflibercept 2 mg	Bimonthly	306	7	51.6 (13.9)	8.9 (14.4)	[9]

PRN = pro re nata, injections are administered on an as-needed basis depending on disease activity;
VA = visual acuity; ETDRS = early treatment for diabetic retinopathy study; SD = standard deviation
