# Supplementary material for: The cost-effectiveness of bevacizumab, ranibizumab and aflibercept for the treatment of age-related macular degeneration—A cost-effectiveness analysis from a societal perspective
Source: PLoS One. 2018 May 17;13(5):e0197670. doi: 10.1371/journal.pone.0197670 (PMC5957378; doi:10.1371/journal.pone.0197670)
Supplement: S3 Table — (PDF) [file pone.0197670.s003.pdf]

S3 Table. Direct and indirect costs for neovascular AMD treatment

	Unit	Costs (€)	SE	Distribution	Source
**Aflibercept**	Per injection<br>Average per year: 7.0	943.48		Fixed	DNHCI
**Ranibizumab**	Per injection<br>Average per year: 6.2	979.14		Fixed	DNHCI
**Bevacizumab**	Per injection<br>Average per year: 7.0	44.45		Fixed	DNHCI
**Medical visit**	Per visit<br>Per year: 12	140.42		Fixed	Radboudumc
**Optical coherence tomography**	Per measurement<br>Per year: 12	33.39	5.0	Gamma	Radboudumc
**Fluorescein angiography**	Per measurement<br>Per year: 1	111.33	10.0	Gamma	Radboudumc
**Visual acuity measurement**	Per measurement<br>Per year: 12	12.19	2.0	Gamma	Radboudumc
**Fundus photograph**	Per measurement<br>Per year: 1	39.0	10.0	Gamma	Radboudumc
**Low vision aids***	If 20/20-20/25	218.24	85.95	Gamma	Low Vision Totaal
(One-time)	If 20/30-20/40	451.69	74.97		
	If 20/50-20/100	1020.00	53.74		
	If ≤20/200	1065.87	73.76		
**Nonmedical costs#**	If 20/20-20/25	1141.69	327.09	Gamma	Brown et al.[20]
(One-time)	If 20/30-20/40	6165.32	1947.80		
	If 20/50-20/100	19477.18	2599.00		
	If ≤20/200	42318.61	3822.44		

SE=Standard error; DNHCI=Dutch National Health Care Institute, www.medicijnkosten.nl, 2014; Radboudumc = Department of Ophthalmology of the Radboudumc.

The medical visit is the same regardless of whether an injection was administered during the visit. Costs for low vision aids and nonmedical costs were determined at the end of the first year based on the final visual acuity.

*Low vision aids includes (electronic) loupes, loupe glasses, filter glasses, loupe lamp, monitor loupes;

#Nonmedical costs include caregivers costs for inside activities of daily living (ADL), outside ADL and transportation costs and residence costs.
